# Yin and Yang of Biofilm Formation and Cyclic di-GMP Signaling of the Gastrointestinal Pathogen *Salmonella enterica* Serovar Typhimurium

**DOI:** 10.1159/000519573

**Published:** 2021-11-12

**Authors:** Agaristi Lamprokostopoulou, Ute Römling

**Affiliations:** ^a^Center of Basic Research, Biomedical Research Foundation of the Academy of Athens, Athens, Greece; ^b^Department of Microbiology, Tumor and Cell Biology, Karolinska Institutet, Stockholm, Sweden

**Keywords:** Cyclic diguanylate monophosphate, *Salmonella* Typhimurium, Biofilm formation, Virulence, Immune response

## Abstract

Within the last 60 years, microbiological research has challenged many dogmas such as bacteria being unicellular microorganisms directed by nutrient sources; these investigations produced new dogmas such as cyclic diguanylate monophosphate (cyclic di-GMP) second messenger signaling as a ubiquitous regulator of the fundamental sessility/motility lifestyle switch on the single-cell level. Successive investigations have not yet challenged this view; however, the complexity of cyclic di-GMP as an intracellular bacterial signal, and, less explored, as an extracellular signaling molecule in combination with the conformational flexibility of the molecule, provides endless opportunities for cross-kingdom interactions. Cyclic di-GMP-directed microbial biofilms commonly stimulate the immune system on a lower level, whereas host-sensed cyclic di-GMP broadly stimulates the innate and adaptive immune responses. Furthermore, while the intracellular second messenger cyclic di-GMP signaling promotes bacterial biofilm formation and chronic infections, oppositely, *Salmonella* Typhimurium cellulose biofilm inside immune cells is not endorsed. These observations only touch on the complexity of the interaction of biofilm microbial cells with its host. In this review, we describe the Yin and Yang interactive concepts of biofilm formation and cyclic di-GMP signaling using *S*. Typhimurium as an example.

## Introduction

Regulation of virulence properties of a microbial organism and its interaction with a potential host is highly dependent on environmental conditions. As has been observed exemplarily by laboratory studies, plate-grown cells of the gastrointestinal pathogen *Salmonella enterica* serovar Typhimurium are hardly virulent, while liquid-grown *S.* Τyphimurium cells readily invade host cells [1], a regulation occurring already at the transcriptional level [2]. Part of this delicate regulation between acute virulence and commensalism/chronic infection is executed by a small molecule whose local or global concentration responds readily to environmental conditions, namely the ubiquitous second messenger cyclic diguanylate monophosphate (cyclic di-GMP) [3, 4, 5, 6]. Cyclic di-GMP is one, and perhaps, the most important member of a larger family of cyclic di- and oligonucleotide second messengers that primarily include the predominantly Gram-positive cyclic di-AMP and the hybrid molecule 3′3′-cyclic AMP-GMP [7, 8]. Recently, the discovery of a broad panel of additional cyclic di- and oligonucleotides has been substantialized including compounds previously only predictively chemically synthesized [9, 10, 11, 12, 13, 14]. Cyclic oligonucleotide second messengers possess a major role in the regulation of the activity of nucleases in CRISPR/Cas-based innate immune response against bacteriophages [14, 15, 16]. On an evolutionary scale, the metazoan viral defense cGAS-STING (cyclic GAMP synthase − stimulator of interferon genes) pathway with the 2′3′-cyclic GMP-AMP analog as the second messenger has its foundation in microbial components [13, 17].

The spatial and temporal intracellular concentration of the second messenger cyclic di-GMP which occurs in over 75% of all bacterial species is adjusted on the single-cell level by a multitude of turnover proteins and receptors [18] and consequently delicately regulates a wide variety of physiological and metabolic traits that channel into acute versus chronic virulence, and sessility versus motility, as well as concomitantly in the promotion of antimicrobial and detergent tolerance and tolerance against the immune response. In this context, cyclic di-GMP can direct fundamental processes such as carbon source catabolism, respiration, cell division, and cell shape by affecting global molecular processes such as RNA turnover, proteolysis, protein acetylation, secretion, and the catalytic activity of biofilm matrix biosynthesis enzymes. This physiological and behavioral consequences have a wide impact not only in the clinical, industrial, and agricultural setting, but also shape the ecology in oceans and affect geochemical relevant global compounds and cycles, such as the denitrification cycle [19, 20, 21, 22, 23]. The conformational flexibility and the ability to form various types of oligomers and few amino acids sufficient to define binding make it challenging to predict the binding sites of cyclic di-GMP [5, 24].

**Fig. 1:**
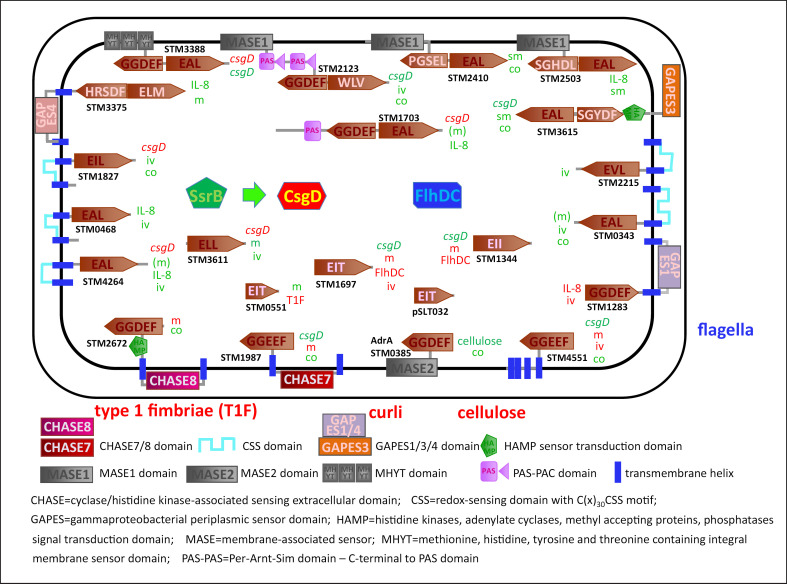
The cyclic di-GMP signaling network of *S.* Typhimurium ATCC14028 and its effect on biofilm formation, motility, and virulence-related phenotypes. The genome of *S.* Typhimurium codes for 22 conserved and evolved cyclic di-GMP turnover proteins. The effect of a gene, as assessed upon deletion, on particular phenotypes (*csgD* = production of the biofilm activator CsgD; cellulose = biosynthesis of the exopolysaccharide cellulose; m = apparent motility; FlhDC = inhibition of the class I flagellar regulon activator FlhD_4_C_2_; IL-8 = secretion of the proinflammatory cytokine IL-8 by the epithelial cell line HT-29; iv = invasion of the intestinal cell line HT-29; sm = survival in macrophages; co = colonization of the gastrointestinal tract (as assessed by analysis of feces)) in the strain *S.* Typhimurium ATCC14028 is indicated; green = promotion of phenotype; red = suppressive effect on phenotype. In brackets, not consistently observed. The response regulator SsrB in its unphosphorylated form activates expression of the *csgD* biofilm regulator gene; FlhDC = class I flagellar regulon activator FlhD_4_C_2_. Cyclic di-GMP, cyclic diguanylate monophosphate; CHASE, cyclase/histidine kinase-associated sensing extracellular domain; CSS, redox-sensing domain with C(x)_30-_CSS motif; GAPES, gammaproteobacterial periplasmic sensor domain; HAMP, histidine kinases, adenylate cyclases, methyl-accepting proteins, phosphatases signal transduction domain; MASE, membrane-associated sensor; MHYT, methionine, histidine, tyrosine, threonine containing integral membrane sensor domain; PAS-PAC, Per-Arnt-Sim domain − C-terminal to PAS domain.

Environmental and intrinsic signals received by cyclic di-GMP turnover proteins determine not only the (acute) virulence properties of microorganisms, but can also provoke the expression of different types of biofilms such as *Pseudomonas aeruginosa* biofilm formation in the urinary tract versus laboratory-grown biofilms [25]. The multitude of signals that direct the turnover activity of the cyclic di-GMP second messenger signaling system (equally as those of other second messengers and phosphotransfer signal transduction systems, chemotaxis systems, and other, which are discussed here in the context of relevant cross talk) on the transcriptional, post-transcriptional, and post-translational level integrates into a specific output response which is equally dependent on the receptor and target proteome status combined with the rest of the proteome [6]. The predominant extracellular matrix components that cover the bacterial cells in a honeycomb-like fashion include amyloid curli fimbriae and the exopolysaccharide cellulose. Curli and (phosphoethanolamine modified) cellulose possess clearly defined features, which point to opposite functionality [27, 28, 29]. In this mini-review, we discuss the Yin and Yang functionality of the extracellular matrix components, biofilm formation, biofilm regulators, and cyclic di-GMP signaling in bacterial and bacterial-host interactions taking mainly the gastrointestinal pathogen *S.* Typhimurium as an example (Fig. 1).

## Amyloid Curli Fimbriae and the Exopolysaccharide Cellulose as Opposing Extracellular Matrix Components of *S.* Typhimurium Biofilms

**Fig. 2:**
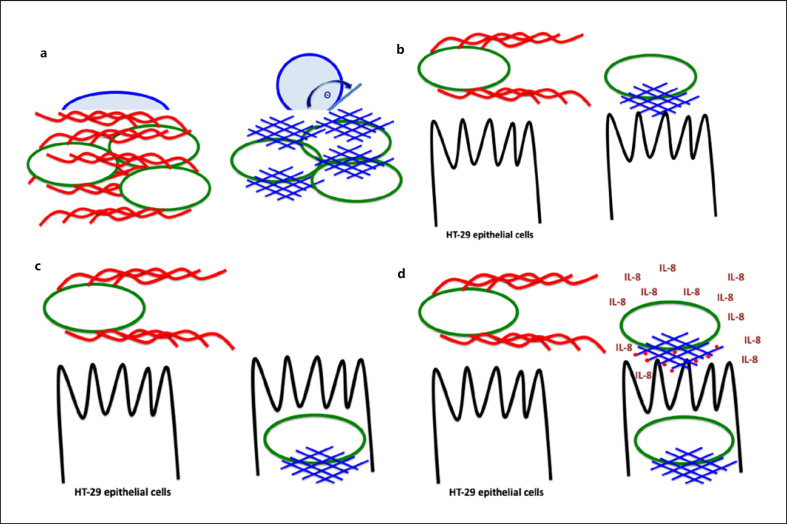
The extracellular matrix components of the rdar biofilm, the exopolysaccharide cellulose, and amyloid curli fimbriae possess distinct features and furnish *S*. Typhimurium cells with a distinct biological function. (**a**) While expression of the exopolysaccharide cellulose provides a hydrophilic cell surface, expression of the amyloid curli fimbriae leads to a more hydrophobic surface as exemplified by assessment of surface tension [31]. A larger contact angle Θ indicates a more hydrophobic surface. Expression of the exopolysaccharide cellulose prevents adhesion (**b**), invasion (**c**), and secretion of the proinflammatory cytokine IL-8 (**d**) of *S.* Typhimurium to the gastrointestinal epithelial cell line HT-29, while expression of the amyloid curli fimbriae promotes adhesion, invasion, and secretion [83, 101, 186]. However, this microbial behavior is context dependent [41]. Rdar, red, dry, and rough.

A highly hydrophobic outer shell encloses cells of the plate-grown rdar (red, dry, and rough) morphotype of *S. Typhimurium*, *Escherichia coli*, and other enterobacteria upon expression of two major extracellular matrix components: amyloid curli fimbriae and the exopolysaccharide cellulose (Fig. 2; [30, 31, 32]). These two polymeric extracellular matrix components tightly interact to display a full biofilm phenotype (bacterial wood), but with each of these matrix components actually to possess a distinct and frequently opposite functionality (Fig. 2). The extracellularly polymerized amyloid curli with subunits characterized by 5 parallel pseudo-repetitive beta-strands converts the cell surface toward hydrophobicity with promiscuous adhesive properties toward proteins and surfaces (Fig. 2a; [31, 33, 34, 35, 36, 37]). Consequently, biofilm cells expressing curli fimbriae interact tightly with abiotic and biotic surfaces [28, 30]. In contrast, expression of the exopolysaccharide cellulose leads to an overall hydrophilic cell surface as measured by the contact angle of bacterial macrocolonies [31]. Cellulose provides predominantly flexible cell-cell interactions in a static rich culture medium, while under continuous flow in minimal medium cellulose contributes to surface adherence and cell-cell interactions [31, 38, 39, 40]. Although the expression of these two matrix components is tightly coupled in plate-grown biofilms through direct and indirect regulation by the bistably expressed transcriptional regulator CsgD, the concomitant expression can be uncoupled under alternative environmental conditions or even subsequent genetic alteration [40, 41]. The distinct physicochemical characteristics of the extracellular biofilm matrix components curli and cellulose extend into distinct host-pathogen interactions as described below.

## The Cyclic di-Nucleotide Second Messenger Signaling System

The universally conserved predominantly bacterial secondary messenger cyclic di-GMP was initially identified in the bacterium *Komagataeibacter xylinus* (originally *Acetobacter* [*Gluconacetobacter*] *xylinum*) to activate the biosynthesis of the exopolysaccharide cellulose [42]. Interactive with other nucleotide signaling systems and upon integration of still unknown signaling pathways, cyclic di-GMP acts as a nearly ubiquitous signal currency to exponentially translate and integrate a multitude of environmental and intracellular signals into the opposite sessility/motility lifestyle behavior concomitant with the cell cycle, cell morphology, metabolism, secondary metabolites, and physiology. The local and global elevation of the cyclic di-GMP signal thus results in the transition from a motile planktonic growth of single cells to an often sessile multicellular biofilm.

The turnover of cyclic di-GMP is controlled by ubiquitous GGDEF and EAL or HD-GYP single or hybrid domain proteins encoded by numerous gene copies in variable numbers and ratios grossly correlated with genome size within a phylum [4, 43, 44, 45]. Thereby, cyclic di-GMP is synthesized from two molecules of GTP by the diguanylate cyclase activity of GGDEF domains and hydrolyzed to linear pGpG or GMP through the phosphodiesterase activity of EAL or HD-GYP domains [46]. Both the N-terminal signaling domains and the catalytic domains can receive regulatory signals which allosterically regulate the synthesis and hydrolysis of the messenger [47, 48]. For example, in the plant pathogen *Agrobacterium tumefaciens*, the level of cyclic di-GMP is controlled by a hybrid GGDEF-EAL protein, DcpA, that confers either diguanylate cyclase or phosphodiesterase activities depending on the absence or presence of the pteridine reductase PruA [49]. Similarly, in *P. aeruginosa*, the GGDEF-EAL protein MucR, confers diguanylate cyclase activity when the bacterium is planktonic while the EAL domain is active conferring a phosphodiesterase function when in a biofilm, with the activity growth responsive to nitric oxide [50]. In *S*. Typhimurium, the hybrid GGDEF-EAL protein STM3388, the homolog of MucR, subsequently represses and activates production of the biofilm regulator CsgD during the growth phase [51].

In the signaling cascade downstream of synthesis, specific effector proteins directly or indirectly mediate the physiological output and phenotypes. Since the first cyclic di-GMP receptor was identified in 1987, namely the cellulose synthase of *Gluconacetobacter xylinus* [52, 53], with subsequent identification of C-terminal PilZ as the binding domain, numerous receptors with distinct cyclic di-GMP binding motifs including RNA aptamers have been elucidated [54, 55, 56].

Despite the wealth of experimental data that address various aspects of the cyclic di-GMP signaling system and its physiological consequences, one major question remains unanswered: how are biofilm extracellular matrix components differentially regulated to promote the various temporal and spatial restricted types of biofilms [57] and do these different types of biofilms display distinct tolerance profiles? For example, the phosphodiesterase BinA of *Vibrio fischeri* adjacent of the Spy exopolysaccharide operon downregulates production of a cellulose-like exopolysaccharide [58]. In *S.* Typhimrurium, the evolved phosphodiesterase STM0551 within the type 1 fimbrial gene cluster represses the adjacent fimbrial genes [59].

## Regulation of Motility versus Sessility by Cyclic di-GMP Signaling

Perhaps the most fundamental feature of cyclic di-GMP is to confer the sessility versus motility lifestyle switch. First demonstrated with model signaling proteins including the diguanylate cyclase AdrA and the phosphodiesterase YhjH, high cyclic di-GMP levels enhance biofilm formation [46, 60], while low cyclic di-GMP levels result in promotion of bacterial motility which can result in a planktonic lifestyle [60, 61, 62]. Cyclic di-GMP ubiquitously regulates sessility versus motility in all investigated bacteria with a multitude of physiological and metabolic adjustments, beyond the simple stimulation of biofilm transcription factors and biosynthesis enzymes that synthesize extracellular matrix components and post-translational downregulation of flagellar-based motility [5, 20, 63]. Such a concomitant adjustment occurs, for example, in *Vibrio cholerae* where high level of cyclic di-GMP promotes DNA repair through the VpsT and VpsR cyclic di-GMP-dependent biofilm regulators. This regulation positively induces expression of the DNA repair gene 3-methyladenine glycosylase (*tag*) offering higher tolerance to DNA damaging conditions [64].

**Fig. 3:**
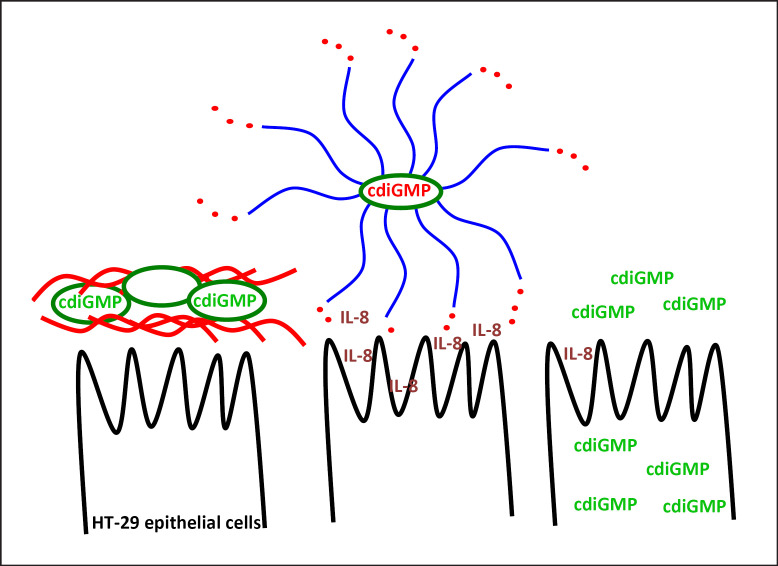
Yin and Yang role of cyclic di-GMP signaling in stimulation of secretion of the pro-inflammatory cytokine IL-8 by *S.* Typhimurium. Left: high intracellular cyclic di-GMP levels stimulate *csgD* expression and production of the extracellular matrix component cellulose. Cells in such a biofilm state do not stimulate production of the pro-inflammatory cytokine IL-8 [69, 83]. Middle: low intracellular cyclic di-GMP levels stimulate secretion of monomeric flagellin by *S.* Typhimurium and IL-8 secretion by HT-29 cells is stimulated [69, 83]. Right: cyclic di-GMP injected into cell lines and (upon the presence of receptors) extracellular cyclic di-GMP stimulates an innate immune response [110, 187, 188]. Cyclic di-GMP, cyclic diguanylate monophosphate.

In *S.* Typhimurium and *E. coli*, the *csgD-*mediated biofilm has been shown to be a major hub of cyclic di-GMP regulation with the orphan response regulator CsgD to promote the transcription of genes encoding biofilm matrix components. These include the *csgBAC* operon of minor and major curli fimbriae subunits and, indirectly, cellulose production by activating the gene for the diguanylate cyclase AdrA [66, 67, 68]. CsgD-mediated biofilms contribute not only to the transmission of *S.* Typhimurium, but also to biofilm formation and persistence in the context of the gastrointestinal tract, plants, and other environments (Fig. 3; [68, 69, 70]). The bistable expression of CsgD ensures different subpopulations of cells with distinct biofilm formation and virulence properties [40, 69], which provide the multicellular cell population with various immediate physiological possibilities.

**Fig. 4:**
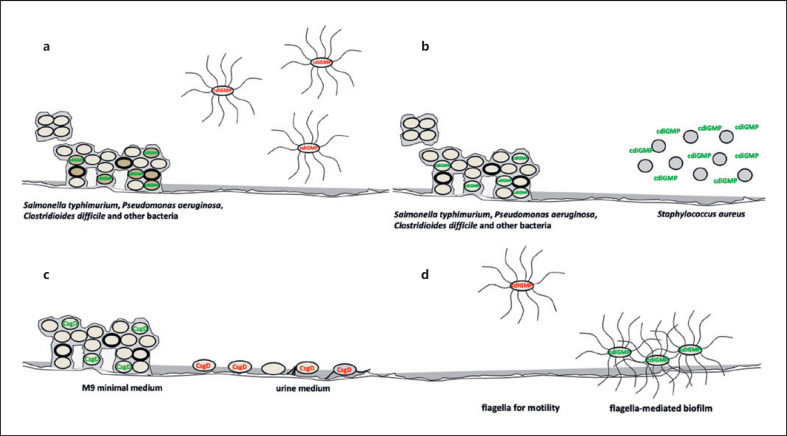
Cyclic di-GMP and biofilm components in different context and its effect on biofilm formation by *S.* Typhimurium. (**a**) Sessility (biofilm formation) is stimulated by local or global high levels of cyclic di-GMP, while motility is stimulated by low levels of cyclic di-GMP in bacteria such as *S.* Typhimurium, *P. aeruginosa*, and *Clostridioides difficile*. (**b**) High intracellular cyclic di-GMP levels trigger biofilm formation of *S.* Typhimurium [46], while extracellularly applied cyclic di-GMP inhibits cell aggregation and biofilm formation by *Staphylococcus aureus* [73]. (**c**) Biofilm formation of *S.* Typhimurium is largely dependent on *csgD* in M9 minimal medium [40], while *csgD* expression inhibits biofilm formation in urine medium on a silicone surface (Wang and Römling, unpublished [78]). (**d**) Flagella promote swimming motility in liquid medium and swarming motility on a surface at low cyclic di-GMP levels, but also can be part of the extracellular biofilm matrix with, in *S.* Typhimurium, cell-associated flagellin to be upregulated upon expression of the diguanylate cyclase AdrA suggesting that production of flagella is increased [46, 83]. Cyclic di-GMP, cyclic diguanylate monophosphate.

With a cyclic di-GMP binding motif absent, *csgD* expression itself is a major target of cyclic di-GMP signaling on the transcriptional and post-transcriptional level (Fig. 1; [71, 72]). In contrast to the ubiquitous intracellular role of cyclic di-GMP, application of cyclic di-GMP extracellularly inhibits biofilm formation in bacteria such as *Staphylococcus aureus* (Fig. 4a; [73]). Indeed, extracellular cyclic di-GMP has been proposed as a treatment option against biofilm diseases; however, the effective mechanisms have been poorly explored.

*S.* Typhimurium and *E. coli* possess an array of fimbriae that can potentially promote biofilm formation [74, 75, 76] expressed under different, and often still undefined, environmental conditions with distinct regulatory schemes. Biofilm development of *S.* Typhimurium in batch cultures demonstrated a type 1 fimbriae and *csgD-*mediated biofilm after 24 h that transforms into a solely *csgD-*mediated biofilm after 48 h [77]. Surprisingly, when grown on a silicone surface mimicking a urinary catheter in the artificial urine medium, *csgD* expression repressed biofilm formation as a *csgD* mutant showed higher biofilm formation as the wild type (Fig. 4b; Xiaoda Wang and Ute Römling, unpublished work; [78]). One explanation is possible repression of alternative biofilm matrix components such as type 1 fimbriae by *csgD*. Another possibility is that adhesive components are surface-selective with deletion of *csgD* to expose silicone-specific adhesins on the cell surface. In this context, the composition of the biofilm of *P. aeruginosa* formed on the surface of the silicone catheter under urinary tract growth conditions has been shown to be fundamentally different from medium-grown biofilms [79]. Motility is commonly negatively regulated by cyclic di-GMP in various bacteria [46, 80]. A wide variety of motility modes are repressed by cyclic di-GMP signaling including flagella-mediated swimming and swarming motility and type IV pili surface motility [81]. In *S.* Typhimurium, post-translational regulation by cyclic di-GMP, which binds to the PilZ domain protein YcgR, leads to a conformational change in the protein [80, 82]. Consequently, cyclic di-GMP loaded YcgR can form a complex with FliG and FliM proteins that are part of the flagella rotor. Although cyclic di-GMP can also inhibit expression of the flagellar regulon cascade in *E. coli*, overexpression of diguanylate cyclases in *S.* Typhimurium enhanced cell-associated flagellin most likely in the form of flagella [83]. This scenario is consistent with the idea that flagella have multiple roles as propellor of motility, as surface sensor and adherence factor, even constituting an extracellular matrix component of biofilms (Fig. 4c; [84, 85]).

## Regulation of Acute versus Chronic Virulence by Cyclic di-GMP Signaling

Cyclic di-GMP signaling regulates virulence of human, animal, and plant pathogens from *S.* Typhimurium and *Mycobacterium tuberculosis*, to the obligate intracellular pathogen *Anaplasma phagocytophilum* and the plant pathogen *Xanthomonas campestris* [5, 9, 26, 86, 87].

Being a major virulence factor in chronic and recurrent infections, acute infections also include temporal and spatial aspects of biofilm formation such as adherence, surface colonization and tolerance against immune components, antimicrobial agents, and detergents [88, 89]. Motility and chemotaxis are required for acute infection processes. Thus, cyclic di-GMP-mediated transition between acute and chronic infection properties is crucial for a successful infection [62, 90]. Remarkable is the intrinsic inconsequence of high bacterial intracellular cyclic di-GMP concentration to lead to a biofilm status, which triggers a low-level immune response, while a low bacterial intracellular cyclic di-GMP level that leads to a virulence status provokes a high immune response. In contrast, host tissue available cyclic di-GMP provokes a substantial innate and adaptive immune response.

## Acute Virulence Phenotypes Regulated by Cyclic di-GMP Signaling

Acute infections are based on short-term expansion of microbes that mostly involve planktonic (and motile) bacterial cells to employ the repertoire of virulence factors to invade and severely damage the tissue and to cause a substantial immune response. During acute infection by *S.* Typhimurium, the microorganisms are hypothesized to predominantly form biofilms in the gastrointestinal lumen with a fraction of cells breaching the epithelial cell lining. Nine cyclic di-GMP turnover proteins contribute to cecum colonization in the microbiota-depleted streptomycin-treated mouse model [91]. The contribution of (predicted) phosphodiesterases such as STM3615 (YhjK) and diguanylate cyclases such as STM2672 (YfiN) and *Salmonella-*specific STM4551 points to a complex role of cyclic di-GMP in persistent gut colonization (Fig. 1; [91]), with STM3615 also deficient in the colonization of mesenteric lymph nodes and the spleen (Lamprokostopoulou, Römling, and W.-D. Hardt, unpublished observations). Although the biofilm regulator *csgD* and curli are expressed in the gastrointestinal tract, the panel of regulatory cyclic di-GMP turnover proteins is distinct compared to regulation of plate-grown biofilms [51, 71, 92]. The putative phosphodiesterase STM3615, though, has an unconventional role in regulation of rdar biofilm formation in the background of deletion of *dsbA dsbB* genes involved in periplasmic disulfide bond formation, with the involvement of the catalytic activity to be tested [93].

Although *S.* Typhimurium causes acute gastroenteritis, the disease is self-limiting in most immune-competent individuals due to the massive immune response combined with neutrophil influx. A particularly invasive *S.* Typhimurium clone, ST313, with enhanced virulence resembling typhoid fever and reduced biofilm formation, has emerged in Africa in HIV and malaria infected individuals and upon malnutrition [94]. The infection process of *S*. Typhimurium is regulated by cyclic di-GMP signaling and biofilm components at various stages as dissected by experimental studies with cell culture and animal models to show unique and distinct contributions of individual cyclic di-GMP turnover proteins (see below; [69, 83, 91, 95, 96, 97]).

Close association with epithelial cells is a characteristic of gastrointestinal pathogens and one of the first contacts of the bacteria with host tissue. *S. Typhimurium* forms biofilms on intestinal epithelial cells [98, 99]. Surprisingly, the two *csgD-*activated extracellular matrix components curli and cellulose have opposing roles in cell adherence. Curli fimbriae promote adhesion, while the exopolysaccharide cellulose inhibits adhesion of *S.* Typhimurium to the gastrointestinal cell line HT-29 (Fig. 2b; [83, 99, 100]). While a similar adherence pattern has been observed in a commensal and urinary tract infection *E. coli* strain [28, 101], the functionality of cellulose is context dependent; in the probiotic strain *E. coli* Nissle 1917, cellulose production promotes adhesion [41].

Invasion or uptake of *S.* Typhimurium into cells of the epithelial cell lining is one of the key steps in the pathogenicity of *S.* Typhimurium [102]. Saturation of the bacterial cell with cyclic di-GMP by overexpression of the diguanylate cyclase AdrA had a profound negative effect on invasion into the gastrointestinal epithelial cell line HT-29 [83]. Inhibition of virulence properties can be partially or even fully restored upon deletion of the biofilm regulator *csgD*, identifying *csgD* as one central hub for the acute virulence versus biofilm switch at the epithelial cell lining in *S.* Typhimurium [83]. Relieve of invasion occurs further through inhibition of the production of cellulose and capsule extracellular matrix components (Fig. 2c; [31, 65, 83]). Cellulose production can be activated, though, by cyclic di-GMP independently of CsgD [31, 103]. Possible mechanisms of reduction of invasion are prevention to establish adherence and shielding the type III secretion system-1 nanomachine via production of the cellulose exopolysaccharide [83, 104].

Dissecting the effect of individual cyclic di-GMP turnover proteins showed that 10 out of 20 deletions of individual GGDEF/EAL domain genes altered the invasion phenotype with 7 mutants showing a conventional and three mutants showing an unconventional phenotype. The molecular basis of interference with invasion has started to become unraveled for some of these GGDEF/EAL domain proteins (see below).

Breaching the epithelial cell lining by *S.* Typhimurium causes massive secretion of the pro-inflammatory cytokine IL-8, which subsequently attracts neutrophils to clear the infection [105]. Again, flooding the bacterial cell with cyclic di-GMP abolishes induction of the pro-inflammatory cytokine IL-8 in the epithelial cell line HT-29 (Fig. 2d, 3; [83]). Deletion of the genes for 6 cyclic di-GMP turnover proteins affects IL-8 secretion, while three of those proteins affect both invasion and IL-8 secretion. In contrast, secreted cyclic di-GMP and cyclic di-GMP systemically applied to the host is commonly immuno­stimmulatory, with cyclic di-GMP recognized as a noncytotoxic adjuvant [17, 83, 106, 107, 108, 109, 110].

**Fig. 5:**
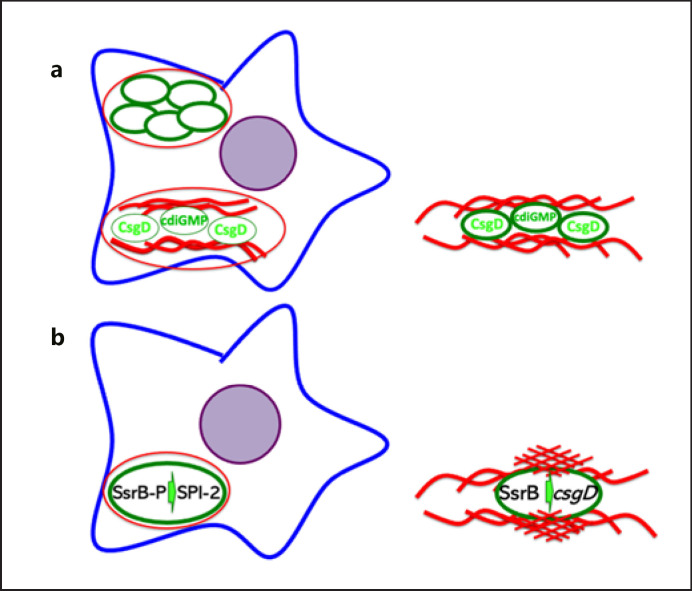
Contribution of biofilm components and virulence factors to virulence and biofilm formation in *S.* Typhimurium. (**a**) Cellulose production and *csgD* expression of *S.* Typhimurium establish extracellular and intracellular biofilms [5, 39. 40, 91, 95, 96]. Left: *S.* Typhimurium produces cellulose inside macrophages. Right: *S.* Typhimurium produces cellulose in extracellular biofilms. (**b**) The phosphorylated SsrB response regulator of the SsrA/SsrB 2-component system stimulates expression of the TTSS-2 2-component system inside the *Salmonella*-containing vacuole of macrophages [131], while the unphosphorylated SsrB response regulator aids promoter activation of the *csgDEFG* operon encoding *csgD*, the rdar biofilm activator, and additional genes required for the biogenesis of amyloid curli fimbriae [120, 132]. Cyclic di-GMP, cyclic diguanylate monophosphate.

Macrophages take up *Salmonella* beyond the gastrointestinal epithelial barrier for transport to inner organs via the blood stream [111, 112, 113]. *S.* Typhimurium uptake and survival in macrophages has been investigated in *Salmonella* susceptible animal models by mimicking *Salmonella enterica* serovar Typhi infection in humans long before the invasive *S. Typhimurium* ST313 clone emerged [69, 114]. *S.* Typhimurium produces cellulose within the *Salmonella*-containing vacuole in macrophages, which restricts its proliferation and attenuates acute virulence during systemic infection of *Salmonella* susceptible mice (Fig. 5a; [39, 96]). Different mechanisms can lead to alteration of cellulose production such as the MtgC virulence factor which interacts with the F_1_F_0_ ATP synthase to restrict cellulose biosynthesis. The cellulase BcsZ, which substantially upregulates virulence of *S.* Typhimurium, suggests that dysregulated biosynthesis of cellulose and not expression of the cellulose synthase BcsA is a determinative virulence modifying factor. Of note, ST313 clone members of *S.* Typhimurium possess a number of single nucleotide polymorphisms inside open reading frames and in intergenic regions, which have been shown to modulate virulence of this organism. For example, an amino acid substitution in the sensory Cache 1 domain of the diguanylate cyclase STM1987 causes reduced cellulose production but enhanced murine and human macrophage survival [97]. Equally, ST313 representatives harbor mutations in the gene for the alkaline phosphatase superfamily member BcsG [115]. As BcsG stabilizes the cellulose synthase BcsA post-translationally in combination with covalent modifications of the glucose subunits growing glucan chain by the phosphoethanolamine phospholipid headgroup [27, 29], the reduced cellulose biosynthesis is predicted to lead to enhanced proliferation in macrophages. Thus, intracellular proliferation without cellulose production opposes growth restriction upon biosynthesis of the cellulose biofilm matrix component, which basically oppositely reflects biofilm formation versus planktonic cell proliferation in the extracellular gastrointestinal space. Whether and, if so, why cellulose-producing bacteria are more susceptible to intracellular antimicrobial defense mechanisms needs to be shown. The channeling of glucose into cellulose instead of glycolysis which is required for *S.* Typhimurium in macrophages might restrict proliferation [116].

In bone-marrow-derived macrophages, at least three different subpopulations of intracellular *Salmonella*: fast, moderate, and slow growing have been identified [95]. Counterintuitively, the cellulose-producing slow-growing subpopulation was significantly more depleted upon deletion of three cyclic di-GMP-specific phosphodiesterases required to lower cyclic di-GMP levels shortly after entry into the macrophages (Fig. 1). The requirement for survival upon immune cell exposure might contribute that *STM3615*, encoding one of two redox-responsive phosphodiesterases, required for proliferation in macrophages, is also required for luminal colonization [91].

Intracellular and extracellular cellulose production provides an excellent example of the distinct roles of biofilms within host cells versus the luminar space. Within host cells, proliferation of planktonic cells is promoted, while slow-growing cells in a cellulose-producing biofilm status might persist without destruction of the host cell on a longer time scale. Outside host cells in the extracellular gastrointestinal space, the large number of biofilm cells outnumbers the few planktonic cells that breach the epithelial cell lining. Whether other biofilm types are expressed in immune cells cannot be excluded.

## Contribution of Type III Secretion System-1

Cyclic di-GMP contributes at many stages to adjust virulence properties of *S.* Typhimurium; however, which of these processes are affected on the molecular level remains unknown. *S.* Typhimurium possesses two type III secretion systems (TTSS-1 and TTSS-2) needle-like nanomachines that tip adhere to the epithelial cell to inject effector proteins for host cell manipulation. Invasion of *S.* Typhimurium into epithelial cells requires genes of the *Salmonella* pathogenicity island 1 that code for the TTSS-1 [117]. TTSS-1 is regulated by a variety of extra- and intracellular signals [118, 119, 120] such as small intestine growth conditions with low oxygen and high salt to promote optimal expression of TTSS-1 proteins. Regulatory pathways for TTSS-1 expression converge at the transcriptional regulator HilA [117]. Polysaccharide components on the surface of bacteria have been demonstrated to interfere with invasion and/or type III secretion system functionality. The length of the O-antigen chain of lipopolysaccharide [121, 122, 123] and likewise in *S.* Typhi, the Vi-capsule [124] counteracts invasion of host cells. Inhibition by extracellular matrix components is, however, not universal as in *P. aeruginosa* biosynthesis of extracellular biofilm matrix does not seem to correlate with interference with type III secretion functionality [90]. On the other hand, in *S.* Typhimurium, adhesive curli fimbriae mediate adherence [28, 83], a prerequisite for TTSS-1 functionality and invasion [125].

In many bacteria including *S.* Typhimurium, TTSS-1/2 systems are subject to regulation by cyclic di-GMP signaling on the transcriptional and post-transcriptional level [119]. Thereby, biofilm formation and virulence properties can be closely linked as expression of type III secretion system components can be upregulated in biofilms [93, 126] and required for the formation of mature biofilms and multicellular behavior [127, 128].

In *S.* Typhimurium, *csgD* and cyclic di-GMP signaling interfere with TTSS-1 functionality downstream of the activity of the TTSS-1 central regulator HilA [6, 83, 91]. Thereby, cyclic di-GMP-mediated *csgD* expression has been exemplarily shown to inhibit the secretion of the TTSS-1 effector SopE2 [83, 91, 128, 129]. In *csgD* competent cell, cyclic di-GMP turnover proteins, diguanylate cyclases, and phosphodiesterases regulate secretion of effector proteins by their scaffold rather than by catalytic activity [71]. Those findings are consistent with results from *P. aeruginosa* where TTSS-mediated cytotoxicity toward the CHO cell line is affected by cyclic di-GMP signaling [90]. The subset of GGDEF/EAL mutants demonstrating alteration in cytotoxicity toward the CHO cell line was only partially overlapping with the subset contributing to virulence in a burn wound mouse model.

## Contribution of Type III Secretion System-2

The TTSS-2 is required for survival and proliferation of *S.* Typhimurium inside the *Salmonella*-containing vacuole [130]. The SsrA-SsrB 2-component system is regulated by the transcriptional regulator HilD, which affects the expression of HilA coordinating the expression of TTSS-1 and TTSS-2. A major transcriptional regulator of the TTSS-2 is the response regulator SsrB phosphorylated by its cognate histidine kinase SsrA [131]. On the other hand, though, in the lumen of the gastrointestinal tract of the nematode *Caenorhabditis elegans* unphosphorylated SsrB is an anti-virulence factor to be required for the activation of transcription of the biofilm regulator *csgD* (Fig. 5b; [132]). The association of biofilm formation with TTSS-2 is more tight than previously thought as the TTSS-2-encoded MerR-like transcriptional regulator MlrB repressess *csgD* expression inside macrophages [133]. These findings showed that biofilm formation is tightly counterregulated with virulence in *S.* Typhimurium by even using the same components. Of note, not only SsrB but also CsgD directs biofilm formation in its unphosphorylated form [72].

## Regulation of the IL-8 Response by Cyclic di-GMP Signaling

In the absence of cellulose production, *S.* Typhimurium can effectively bind to and invade epithelial cells via curli fimbriae and subsequently trigger production of the pro-inflammatory cytokine via curli-bound flagellin (Fig. 2d; [37, 56, 101]). High levels of cyclic di-GMP, though, did not trigger IL-8 production by HT-29 cells [83]. Stimulation of secretion of the pro-inflammatory cytokine IL-8 is recovered upon deletion of *csgD*, which relieves the secretion of monomeric flagellin. The non-stimulatory phenotype of *S.* Typhimurium during high cyclic di-GMP concentrations may possibly be a result of inhibition of the secretion of monomeric flagellin inducing IL-8 in the HT-29 cell line [134].

Similar to invasion, the cyclic di-GMP signaling system regulates flagellin secretion, as monitored by stimulation of the secretion of the pro-inflammatory cytokine IL-8, by a complex network of cyclic di-GMP turnover proteins (Fig. 1, 3; [91]). While the diguanylate cyclase STM1287 conventionally represses IL-8 secretion, the two EAL domain proteins, STM0468 and STM4264, and the GGDEF-EAL proteins, STM1703 and STM2503, stimulate IL-8 secretion equally as the degenerated GGDEF-EAL protein STM3375. Several of the EAL proteins seem to work in the same pathway as double mutants do not additively diminish the phenotype.

## Flagella Regulon-Related Phenotypes Affected by Cyclic di-GMP Signaling

With the flagellar regulon cascade delicately manipulated on different levels, the flagellum is not only a bacterial virulence factor with swimming and swarming motility to promote colonization and tissue invasion [83, 136]. Monomeric flagellin is recognized as a major pathogen-associated molecular pattern (PAMP) and systemic antigen being a major antigen in Crohn's disease [136, 137].

Differential in vivo affinities for cyclic di-GMP for the flagellar motor break, the cyclic di-GMP receptor YcgR (2 μM), and subsequently the cellulose synthase BcsA (8 μM) involved in the inhibition of bacterial motility and in increase in cellulose-based biofilm matrix production, respectively, ensure coordinated steps toward *S.* Typhimurium biofilm formation [135, 138]. During bacterial infection, the polymeric flagellar filament can act as a virulence factor with secreted monomeric flagellin as an immunogen triggering innate as well as adaptive host response.

Recognition of flagellin monomers by epithelial cells occurs by pattern recognition receptors. Toll-like receptors (TLRs) are a group of important transmembrane pattern recognition receptors and until now, 15 TLRs have been identified, from which TLR 1–10 are found in humans [139]. TLRs have been found to reside on the surface or within cell compartments of not only epithelial and innate immune cells, but also neuronal cells, endothelial cells, and other cell types. After recognition of PAMPs, TLRs trigger a signaling cascade, which leads to the release of pro-inflammatory cytokines in order to subsequently promote an immune response. Recognition of flagellin by TLR5 and in the case of plants by FLS2 [140] subsequently leads to NF-kB activation, chemokine release, T-cell activation, and other inflammatory phenotypes with flagella production to be shut off at the later stage of infection [141, 142]. In bacterial infection of plants, high intracellular cyclic di-GMP concentrations drastically reduce the virulence of *Pseudomonas syringae* pv. *tomato* (Pto) DC3000 through inhibition of flagellar motility among other pleiotropic effects resulting from cyclic di-GMP signaling on bacterial behavior [143, 144]. Stimulation of the secretion of monomeric flagellin has been observed in response to host cells [82], and it remains to be shown whether this stimulation involves a cyclic di-GMP signaling pathway. Equally whether, and how, secretion of monomeric flagellin is coupled to the flagella biosynthesis process is unknown [128]. In conclusion, the above described work shows that secretion of monomeric flagellin is dependent on the expression of biofilm regulators and cyclic di-GMP signaling [83, 91].

The two evolved EAL domain only proteins STM1344 and STM1697 do not possess phosphodiesterase activity nor do these proteins bind cyclic di-GMP but inhibit the flagella regulon by inhibiting the activity of the class 1 regulator FlhD_2_C_4_ through protein-protein interactions [145, 146, 147]. In this way, STM1344 and STM1697, both contribute to regulation of swimming motility and phase variation of flagellar expression [145, 146]. Both proteins promote virulence presumably by their contribution to the delicate regulation of expression of flagellar antigenic filaments. Furthermore, STM1344 promotes resistance to *Salmonella-*induced oxidative stress and inhibits rapid macrophage killing [148].

On the other hand, the EAL-only protein YhjH is the only motility-dedicated phosphodiesterase [92, 135]. YhjH, despite possessing catalytic activity, is actually more closely related to STM1344 and STM1697 than to any other EAL domain in *S.* Typhimurium [67, 149]. Three diguanylate cyclases differentially feed into the inhibition of motility addressing the YcgR motor break (STM2672), the BcsA cellulose synthase (STM1987), or both receptors (STM4551) [135].

## Bacterial Cyclic di-GMP Signaling in Immunity

Overgrowth of the microbial flora is prevented by an outer and inner mucus layer on the surface of the epithelium [150], which provides a mechanical, physicochemical, and biological barrier accumulating bacteriolytic enzymes like lysozyme and antimicrobial peptides secreted from Paneth cells [151, 152]. In addition, nutritional immunity challenges, for example, iron acquisition by microbial produced siderophores [153, 154]. Microbial secreted cyclic di-GMP can contribute not only to stimulate innate immunity, but to overcome nutritional immunity [12, 109]. Upon ingestion, few *S.* Typhimurium cells penetrate the mucus layer to reach the mucosal cell lining as the first barrier [155]. In mammalian as well as in plant host cells, innate immune receptors located in the cell membrane and intracellular receptors recognize PAMPs and induce an innate immune response known as pattern-triggered immunity (PTI) as a first line of response [156, 157]. Commensal bacteria trigger low-level PTI, evade, or even suppress PTI in order to successfully colonize the host [158]. Pathogen-PAMPs include the following: lipid A part of the lipopolysaccharide present in the outer membrane of Gram-negative bacteria, components of the bacterial cell wall such as peptidoglycan, microbial DNA, and physiological amyloids such as curli [159]. Another PAMP that plays an important role in triggering mucosal innate immune responses, as described above, is flagellin [160].

Based on initial reports [106, 161], cyclic di-GMP was recognized as a PAMP, to trigger protective host innate and adaptive immune responses [110]. The comprehensive stimulation of immunity might contribute cyclic di-GMP to be delivered exogenously in a murine model of bacterial pneumonia. A local or systemic administration of cyclic di-GMP prior to challenge with *Klebsiella pneumoniae* resulted in significantly increased animal survival and bacterial reduction in the lung and blood [110]. In combination with the initiation of robust innate and adaptive immune responses characterized by enhanced accumulation of neutrophils and alphabeta T cells, as well as activated natural killer cells and macrophages expressing inducible nitric oxide synthase and nitric oxide, the cell recruitment was associated with early elevated expression of chemokines and type I cytokines. These initial fundamental findings established cyclic di-GMP and subsequently analogous cyclic di-nucleotides not only as effective immune-modulators and enhancers, but also as potential anti-biofilm and anticancer agents. The subsequent identification of cyclic di-GMP and other cyclic di-nucleotide receptors in mammals paved the way for the identification of the central cGAS-cGAMP-STING axis for sensing and responding to cytoplasmic nucleic acids [162, 163, 164]. Cyclic di-GMP can also activate the intracellular sensor STING, suggesting that cytosolic bacteria release this immune activator [38, 165]. Upon *Salmonella* infection and subsequent cyclic di-GMP release, STING activation induces Interferon Regulatory Factor 1 responsible for TH17 subspecification in the mucosal immune system [166]. The association between high cyclic di-GMP concentration in the colon and stabilization of STING by cyclic di-GMP-induced ubiquitination in a mouse model of spontaneous colitis indicates an underestimated degree of interkingdom cross talk by this ubiquitous second messenger [167]. These observations in an animal model also put forward that substantial secretion of cyclic di-GMP and other cyclic di-nucleotides can occur, perhaps not only by gastrointestinal bacteria such as *E. coli*, but also lung pathogens like *M. tuberculosis* [168, 169].

## The Yin and Yang of Biofilm Formation in the Gastrointestinal Tract

**Fig. 6:**
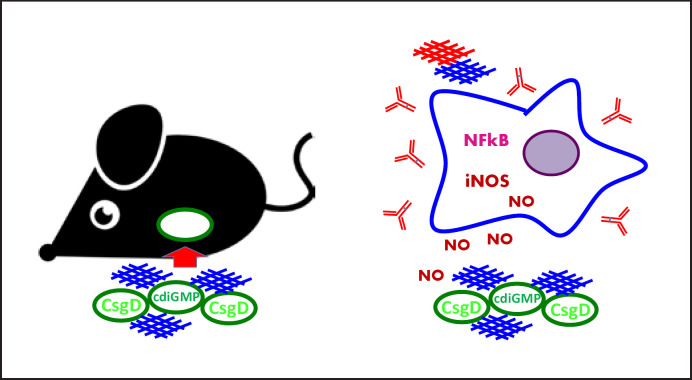
Contribution of the biofilm component curli to intestinal barrier function and invasion versus autoimmunity, seed function, and inflammation. Luminally expressed curli are recognized by TLR2/TLR1 in combination with CD14. This receptor recognition of amyloid fibers induces a phosphatidylinositol 3-kinase-dependent pathway to strengthen the epithelial barrier function in order to prevent invasion of cells into deeper tissue (left). Amyloid fibers or bacterial cells expressing curli located systemically or exposed to immune cells cause the production of autoimmune antibodies, enhance amyloid formation, and cause an inflammatory response via iNOS, NFkappaB, and other pathways. Mouse figure is taken from https://www.flaticon.com/free-icon/mouse-black-animal_40508. TLR, toll-like receptor; cyclic di-GMP, cyclic diguanylate monophosphate.

The biofilm regulator *csgD* is expressed in the gastrointestinal tract and can be required for colonization (Fig. 6; [170, 171]). Consequently, curli (and other physiological microbial amyloids) are produced during acute and chronic infections [172, 173, 174, 175]. These amyloid PAMPs are recognized by TLR1/TLR2 in combination with the CD14 adaptor [176, 177, 178] and intracellular NOD-like receptors [179]. Thereby, recognition of curli by host immune components leads, on the one hand, to the strengthening of the epithelial barrier function and dampens inflammation in the gut [180, 181] and, on the other hand, upon breaching of the intestinal barrier, to autoantibody formation with delayed onset of autoimmunity, inflammation, and functioning as a seed to promote enhanced aggregation leading to neurogenerative diseases [173, 182, 183, 184, 185]. The *csgD* biofilm activator is impaired or absent in the invasive *S.* Typhimurium clone ST313, equally as in *S*. Typhimurium [94]. The deficiency to produce curli may contribute to enhanced translocation as demonstrated for non-ST313 curli mutants [181]. Thus, in the luminar space, production of these physiological amyloids prevents their own, other amyloids and bacterial cell translocation to protect the host from systemic disease and overshooting inflammatory processes.

## Conclusion

Although biofilms are commonly considered as one physiological state in a bacterium, various modes of biofilm formation exist that might have different consequences on microbial host interactions. Thereby, even concomitantly expressed extracellular matrix components can play opposite roles in microbial physiology. The major regulator of biofilm formation, the ubiquitous second messenger cyclic di-GMP, delicately regulates biofilm formation and pathogen-host interactions. Thereby, extracellularly of bacteria, the role of cyclic di-GMP and other cyclic di-nucleotides in a host environment is in stark contrast to the intracellular role of cyclic di-GMP in bacteria. Cyclic di-GMP is an intracellular second messenger signaling molecule in bacteria that promotes biofilm formation, which transforms cells into a low virulence, (relatively) low immunogenic status that is more similar to persistent commensalism than reflecting an acutely virulent pathogen. However, microbial secreted or systemically and mucosally applied cyclic di-GMP, cyclic di-AMP, and other cyclic di-nucleotides either stimulate or also inhibit immune responses. In this way, bacteria have the possibility to distinctively manipulate the immune system response. To what extent this secretion process occurs in bacteria, whether and how it is regulated, and in the case of secretion of cyclic di-nucleotides by *E. coli* and *M. tuberculosis*, its precise molecular mechanisms need to be unraveled. Furthermore, the interaction of biofilms with immune cells such as M cells and dendritic cells at the interface between innate and adaptive immune response with cyclic di-nucleotide-producing bacteria has not been thoroughly explored.

## Conflict of Interest Statement

The authors have no conflicts of interest to declare.

## Funding Sources

Work by the authors described in this review has been supported by the Swedish Research Council, Scientific Council for Natural Sciences and Engineering, the Marie Curie Innovative Training Network “EUs IMO-Train,” and the Karolinska Institutet.

## Author Contributions

Agaristi Lamprokostopoulou and Ute Römling drafted the review.
